# Circulating sex hormones in relation to anthropometric, sociodemographic and behavioural factors in an international dataset of 12,300 men

**DOI:** 10.1371/journal.pone.0187741

**Published:** 2017-12-27

**Authors:** Eleanor L. Watts, Paul N. Appleby, Demetrius Albanes, Amanda Black, June M. Chan, Chu Chen, Piera M. Cirillo, Barbara A. Cohn, Michael B. Cook, Jenny L. Donovan, Luigi Ferrucci, Cedric F. Garland, Graham G. Giles, Phyllis J. Goodman, Laurel A. Habel, Christopher A. Haiman, Jeff M. P. Holly, Robert N. Hoover, Rudolf Kaaks, Paul Knekt, Laurence N. Kolonel, Tatsuhiko Kubo, Loïc Le Marchand, Tapio Luostarinen, Robert J. MacInnis, Hanna O. Mäenpää, Satu Männistö, E. Jeffrey Metter, Roger L. Milne, Abraham M. Y. Nomura, Steven E. Oliver, J. Kellogg Parsons, Petra H. Peeters, Elizabeth A. Platz, Elio Riboli, Fulvio Ricceri, Sabina Rinaldi, Harri Rissanen, Norie Sawada, Catherine A. Schaefer, Jeannette M. Schenk, Frank Z. Stanczyk, Meir Stampfer, Pär Stattin, Ulf-Håkan Stenman, Anne Tjønneland, Antonia Trichopoulou, Ian M. Thompson, Shoichiro Tsugane, Lars Vatten, Alice S. Whittemore, Regina G. Ziegler, Naomi E. Allen, Timothy J. Key, Ruth C. Travis

**Affiliations:** 1 Cancer Epidemiology Unit, Nuffield Department of Population Health, University of Oxford, Oxford, United Kingdom; 2 Division of Cancer Epidemiology and Genetics, U.S. National Cancer Institute, Bethesda, MD, United States of America; 3 Department of Epidemiology and Biostatistics, University of California-San Francisco, San Francisco, CA, United States of America; 4 Department of Urology, University of California-San Francisco, San Francisco, CA, United States of America; 5 Program in Epidemiology, Fred Hutchinson Cancer Research Center, Seattle, WA, United States of America; 6 Child Health and Development Studies, Public Health Institute, Berkeley, CA, United States of America; 7 School of Social and Community Medicine, University of Bristol, Bristol, United Kingdom; 8 Intramural Research Program, National Institute on Aging, Bethesda, MD, United States of America; 9 Department of Family Medicine and Public Health, University of California, San Diego, CA, United States of America; 10 Cancer Epidemiology Centre, Cancer Council Victoria, Melbourne, Victoria, Australia; 11 Centre for Epidemiology and Biostatistics, Melbourne School of Population and Global Health, The University of Melbourne, Victoria, Australia; 12 SWOG Statistical Center, Fred Hutchinson Cancer Research Center, Seattle, WA, United States of America; 13 Division of Research, Kaiser Permanente Northern California, Oakland, CA, United States of America; 14 Department of Preventive Medicine, Norris Comprehensive Cancer Center, Keck School of Medicine, University of Southern California, Los Angeles, CA, United States of America; 15 School of Clinical Sciences, Faculty of Health Science, University of Bristol, Bristol, United Kingdom; 16 Division of Cancer Epidemiology, German Cancer Research Centre, Heidelberg, Germany; 17 Department of Health, National Institute for Health and Welfare, Helsinki, Finland; 18 University of Hawaii Cancer Center, University of Hawaii, Honolulu, HI, United States of America; 19 Department of Public Health, University of Occupational and Environmental Health, Kitakyushu, Japan; 20 Finnish Cancer Registry, Institute for Statistical and Epidemiological Cancer Research, Helsinki, Finland; 21 Department of Oncology, Helsinki University Central Hospital, Helsinki, Finland; 22 Department of Neurology, University of Tennessee Health Science Center, Memphis, TN, United States of America; 23 Japan-Hawaii Cancer Study, Kuakini Medical Center, Honolulu, HI, United States of America; 24 Department of Health Sciences, University of York, York, United Kingdom; 25 Division of Urologic Oncology, University of California San Diego Moores Cancer Center, San Diego, CA, United States of America; 26 Department of Epidemiology, Julius Center for Health Sciences and Primary Care, University Medical Center Utrecht, Netherlands; 27 MRC-PHE Centre for Environment and Health, Department of Epidemiology and Biostatistics, School of Public Health, Imperial College, London, United Kingdom; 28 Department of Epidemiology, Johns Hopkins University Bloomberg School of Public Health, Baltimore, MD, United States of America; 29 School of Public Health, Imperial College London, London, United Kingdom; 30 Department of Clinical and Biological Sciences, University of Turin, Orbassano, Italy; 31 Unit of Epidemiology, Regional Health Service ASL TO3, Grugliasco, Italy; 32 Biomarkers Group, International Agency for Research on Cancer, Lyon, France; 33 Epidemiology and Prevention Group, Center for Public Health Sciences, National Cancer Center, Tokyo, Japan; 34 Cancer Prevention Program, Fred Hutchinson Cancer Research Center, Seattle, WA, United States of America; 35 Division of Reproductive Endocrinology and Infertility, Keck School of Medicine of the University of Southern California, Los Angeles, CA, United States of America; 36 Departments of Nutrition and Epidemiology, Harvard University T.H. Chan School of Public Health, Boston, MA, United States of America; 37 The Channing Division of Network Medicine, Harvard Medical School, Boston, MA, United States of America; 38 Department of Surgical and Perioperative Sciences, Urology and Andrology, Umeå University, Umeå, Sweden; 39 Department of Clinical Chemistry, Medicum, University of Helsinki, Helsinki, Finland; 40 Department of Diet, Genes and Environment, The Danish Cancer Society Research Center, Copenhagen, Denmark; 41 Hellenic Health Foundation, Athens, Greece; 42 WHO Collaborating Center for Nutrition and Health, Unit of Nutritional Epidemiology and Nutrition in Public Health, Department of Hygiene, Epidemiology and Medical Statistics, School of Medicine, National and Kapodistrian University of Athens, Greece; 43 CHRISTUS Medical Center Hospital, San Antonio, TX, United States of America; 44 Department of Public Health, Norwegian University of Science and Technology, Trondheim, Norway; 45 Department of Health Research and Policy, Stanford University School of Medicine, Stanford, CA, United States of America; 46 Department of Biomedical Data Science, Stanford University, Stanford, CA, United States of America; 47 Clinical Trial Service Unit and Epidemiological Studies Unit, Nuffield Department of Population Health, University of Oxford, Oxford, United Kingdom; Shanghai Diabetes Institute, CHINA

## Abstract

**Introduction:**

Sex hormones have been implicated in the etiology of a number of diseases. To better understand disease etiology and the mechanisms of disease-risk factor associations, this analysis aimed to investigate the associations of anthropometric, sociodemographic and behavioural factors with a range of circulating sex hormones and sex hormone-binding globulin.

**Methods:**

Statistical analyses of individual participant data from 12,330 male controls aged 25–85 years from 25 studies involved in the Endogenous Hormones Nutritional Biomarkers and Prostate Cancer Collaborative Group. Analysis of variance was used to estimate geometric means adjusted for study and relevant covariates.

**Results:**

Older age was associated with higher concentrations of sex hormone-binding globulin and dihydrotestosterone and lower concentrations of dehydroepiandrosterone sulfate, free testosterone, androstenedione, androstanediol glucuronide and free estradiol. Higher body mass index was associated with higher concentrations of free estradiol, androstanediol glucuronide, estradiol and estrone and lower concentrations of dihydrotestosterone, testosterone, sex hormone-binding globulin, free testosterone, androstenedione and dehydroepiandrosterone sulfate. Taller height was associated with lower concentrations of androstenedione, testosterone, free testosterone and sex hormone-binding globulin and higher concentrations of androstanediol glucuronide. Current smoking was associated with higher concentrations of androstenedione, sex hormone-binding globulin and testosterone. Alcohol consumption was associated with higher concentrations of dehydroepiandrosterone sulfate, androstenedione and androstanediol glucuronide. East Asians had lower concentrations of androstanediol glucuronide and African Americans had higher concentrations of estrogens. Education and marital status were modestly associated with a small number of hormones.

**Conclusion:**

Circulating sex hormones in men are strongly associated with age and body mass index, and to a lesser extent with smoking status and alcohol consumption.

## Introduction

Sex hormones in men are fundamental to many aspects of physiology. In the prostate, androgens modulate cell proliferation, differentiation and apoptosis and have been theorized to be involved in the etiology of prostate cancer. Although epidemiological studies have found no consistent association between circulating sex hormone concentrations and prostate cancer risk[1], nearly all metastatic prostate tumors overexpress deregulated androgen receptors[2] and androgen deprivation therapy is a common treatment for metastatic prostate cancer. Genome-wide association studies have also identified single nucleotide polymorphisms associated with prostate cancer risk that interact with, or are localized to, androgen receptors[3, 4]. In addition to their role in prostate cancer, sex hormones have been implicated in the development of other common health outcomes, including hypertension, obesity, diabetes, cardiovascular and renal disease[5–7]. By understanding the determinants of sex hormone concentrations, we may better comprehend disease etiology and the mechanisms of disease-risk factor associations.

The Endogenous Hormones, Nutritional Biomarkers and Prostate Cancer Collaborative Group (EHNBPCCG) was established to conduct collaborative pooled analyses of endogenous hormones and nutritional biomarkers in relation to subsequent prostate cancer risk. This large dataset also provides the opportunity to investigate the cross-sectional associations of anthropometric, sociodemographic, behavioural and other factors with circulating sex hormone concentrations. Although many of these associations have been previously studied[8–12], this collaborative dataset enables robust analyses to be performed for a range of hormones and participant characteristics using unified methods. The large size of this dataset also provides greater power to investigate the associations at extremes of the distribution for known relationships, as well as the opportunity to examine novel associations.

## Subjects and methods

### Data collection

Principal investigators were invited to join the EHNBPCCG if they had published studies on prostate cancer risk and circulating concentrations of sex hormones and/or nutritional biomarkers from blood samples collected prior to the diagnosis of prostate cancer. These were identified using literature search methods described previously[1]. Collaborators provided data on circulating concentrations for up to seven different sex hormones and sex hormone-binding globulin (SHBG) (Table 1) and a wide range of anthropometric, sociodemographic, behavioural and medical factors.

**Table 1:** Assay methods and geometric mean sex hormone and SHBG concentrations.

	Androstenedione (nmol/L)			A-diol-G (nmol/L)			DHEAS (nmol/L)			Testosterone (nmol/L)			DHT (nmol/L)				Estradiol (pmol/L)			Estrone (pmol/L)			SHBG (nmol/L)		
	Method	CV %	Geometric mean (95% CI)	Method	CV %	Geometric mean (95% CI)	Method	CV %	Geometric mean (95% CI)	Method	CV (%)	Geometric mean (95% CI)	Method	CV %		Geometric mean (95% CI)	Method	CV %	Geometric mean (95% CI)	Method	CV %	Geometric mean (95% CI)	Method	CV %	Geometric mean (95% CI)
ATBC	E RIA	7†	4.7 (4.4–5.0)	E RIA	12.3†	5.7 (5.3–6.2)	E RIA	11.3†	3069 (2825–3335)	E RIA	5.5†	20.5 (19.5–21.5)	E RIA		8.9†	1.8 (1.7–2.0)	E RIA	13.7†	102 (97–107)	E RIA	14.1†	153 (146–159)	IRMA	4.2†	85.2 (80.3–90.4)
BLSA	-	-	-	-	-	-	NE RIA	2–6.6‡	3393 (3006–3830)	NE RIA	3.3–6.4‡	15.3 (14.2–16.4)	-		-	-	-	-	-	-	-	-	NE RIA	1.8–22‡	81.1 (74.5–88.3)
CARET	E RIA	5–9‡	2.7 (2.5–2.8)	NE RIA	1.5–4.8‡	12.0 (11.3–12.8)	NE RIA	0.8–1.9‡	1919 (1785–2064)	E RIA	1–12‡	14.1 (13.5–14.7)	-		-	-	E RIA	5–13‡	194 (186–201)	-	-	-	IRMA	2–7‡	27.1 (25.8–28.6)
CHDS	-	-	-	-	-	-	-	-	-	E RIA	9–11^a^	22.0 (21.2–22.8)	-		-	-	E RIA	8–10^a^	162 (156–167)	-	-	-	IA	4–6^a^	32.9 (31.5–34.4)
EPIC	NE RIA	3.5–11.1†	4.7 (4.5–4.8)	NE RIA	4.1–9.9†	12.9 (12.3–13.5)	-	-	-	NE RIA	10.8–14.8†	15.7 (15.3–16.2)	-		-	-	-	-	-	-	-	-	IRMA	7.7–12.2†	42.8 (41.2–44.4)
EPIC Norfolk	-	-	-	N/S		12.1 (11.6–12.6)	N/S		2378 (2268–2493)	N/S		15.2 (14.7–15.6)	-		-	-	-	-	-	-	-	-	N/S		42.0 (40.6–43.5)
FMC	NE RIA	9.5–11.7 ^a^	5.9 (5.6–6.2)	-	-	-	-	-	-	NE RIA	4.5–7.2^a^	22.9 (21.9–23.9)	-		-	-	-	-	-	-	-	-	IMF	6.6–8.7 ^a^	50.7 (48.1–53.4)
HHS	-	-	-	-	-	-	-	-	-	IMF	5.5–13‡	19.7 (18.9–20.6)	-		-	-	-	-	-	-	-	-	IMF	1.3–10.1‡	50.2 (47.6–52.9)
HPFS	-	-	-	NE RIA	6.7†	10.7 (10.2–11.2)	-	-	-	ECIA	4.9†	15.4 (15.0–15.9)	NE RIA		9.7†	1.2 (1.2–1.3)	NE RIA	5.2†	111 (108–114)	-	-	-	IRMA	10.7†	56.9 (55.0–58.9)
JACC	-	-	-	-	-	-	-	-	-	NE RIA	5–12^b^	15.6 (14.4–16.8)	-		-	-	-	-	-	-	-	-	IRMA	5.6–6.9 ^b^	43.5 (39.7–47.6)
Janus	-	-	-	-	-	-	-	-	-	E RIA	5–15‡	22.4 (21.9–22.9)	-		-	-	-	-	-	-	-	-	Precipitation	5–15‡	49.5 (48.2–50.8)
JHCS 1988	-	-	-	-	-	-	-	-	-	-	-	-	-		-	-	E RIA	9–15‡	66 (62–71)	E RIA	9–15‡	109 (102–116)	Precipitation	5–15‡	37.1 (33.9–40.6)
JHCS 1996	E RIA	5–15‡	4.9 (4.5–5.3)	E RIA	5–15‡	9.0 (8.1–9.9)	-	-	-	E RIA	5–15‡	17.4 (16.3–18.6)	E RIA		5–15‡	1.8 (1.6–2.0)	-	-	-	-	-	-	-	-	-
JPHC	-	-	-	-	-	-	-	-	-	ECIA	1–3‡	15.7 (15.1–16.3)	-		-	-	-	-	-	-	-	-	IRMA	2–8‡	47.5 (45.4–49.7)
KPMCP	-	-	-	-	-	-	NE RIA	N/S	2467 (2263–2690)	-	-	-	-		-	-	-	-	-	-	-	-	-	-	-
MCCS	NE RIA	3.3^b^	3.6 (3.5–3.7)	NE RIA	4.3^b^	13.8 (13.3–14.3)	CIA	12.4^b^	2812 (2704–2924)	ECIA	1.6^b^	15.4 (15.0–15.8)	-		-	-	ECIA	11.1 ^b^	107 (105–109)	-	-	-	IA	6 ^b^	36.6 (35.6–37.6)
MEC	-	-	-	NE RIA	3.4†	9.5 (9.2–9.9)	-	-	-	E RIA	3.5†	18.3 (17.8–18.7)	E RIA		3.8†	1.9 (1.9–2.0)	-	-	-	-	-	-	ECIA	3†	35.9 (34.9–37.0)
MMAS	E RIA	8.6–10‡	2.9 (2.8–3.0)	-	-	-	NE RIA	4.1–8.9‡	5375 (5116–5647)	E RIA	4.6–7.2‡	16.4 (15.9–16.9)	NE RIA		10.9–12.2‡	0.7 (0.7–0.8)	NE RIA	3.6–7.1‡	140 (136–143)	-	-	-	Filtration assay	8–10.9‡	30.4 (29.4–31.5)
NSHDC androg	-	-	-	N/S		11.7 (11.1–12.4)	-	-	-	N/S		19.6 (18.9–20.4)	-		-	-	-	-	-	-	-	-	IRMA	N/S	41.1 (39.2–43.1)
NSHDC E_2_	-	-	-	-	-	-	-	-	-	-	-	-	-		-	-	N/S	-	77 (74–79)	-	-	-	-	-	-
PCPT	E RIA	6.9–8.6 ^a^	2.0 (1.9–2.1)	NE RIA	2.7–14.0‡	12.0 (11.6–12.4)	-	-	-	ECIA	7.6–11.9 ‡	12.4 (12.2–12.7)	-		-	-	E RIA	10–14.9‡	118 (116–121)	E RIA	9–15.2 ‡	158 (155–161)	ECIA	5.2–12.2‡	36.6 (35.6–37.6)
PHS	-	-	-	NE RIA	7.6†	13.5 (12.7–14.3)	-	-	-	NE RIA	8.7†	16.0 (15.4–16.6)	E RIA		5.3†	1.2 (1.1–1.3)	-	-	-	E RIA	10†	122 (118–126)	IRMA	8.9†	20.6 (19.6–21.6)
PLCO	NE RIA	14†	4.1 (4.0–4.2)	NE RIA	11†	14.2 (13.7–14.8)	-	-	-	NE RIA	14†	15.9 (15.5–16.3)	-		-	-	-	-	-	-	-	-	IRMA	18†	43.9 (42.6–45.2)
ProtecT	-	-	-	-	-	-	-	-	-	EIA	N/S	13.9 (13.4–14.3)	-		-	-	-	-	-	-	-	-	EIA	-	37.2 (35.8–38.7)
RBS	-	-	-	-	-	-	NE RIA	3.1–7.3‡	2054 (1910–2208)	E RIA	4.1–10‡	10.4 (9.9–10.8)	E RIA		7.5‡	1.4 (1.3–1.5)	E RIA	8–12‡	72 (70–75)	-	-	-	-	-	-

Abbreviations: A-diol-G = Androstanediol glucuronide; ATBC = The Alpha-Tocopherol, Beta-Carotene Cancer Prevention Study; BLSA = The Baltimore Longitudinal Study of Aging; CARET = The Carotene and Retinol Efficacy Trial; CHDS = Child Health and Development Studies; CI = confidence interval; CIA = competitive immunoassay; CV = coefficient of variation; DHEAS = Dehydroepiandrosterone sulfate; DHT = Dihydrotestosterone; E RIA = extraction radioimmunoassay; ECIA = electrochemiluminescence immunoassay; EIA = enzyme immunoassay; EPIC = European Prospective Investigation into Cancer and Nutrition; FMC = Finnish Mobile Clinic Health Examination Survey; HHS = Helsinki Heart Study; HPFS = Health Professionals Follow-Up Study; IA = immunoassay; IMF = immunofluorometry; IRMA = immunoradiometric assay; JACC = Japan Collaborative Cohort Study; JPHC = Japan Public Health Center-based Prospective Study; JHCS = Japan-Hawaii Cancer Study; KPMCP = Kaiser Permanente Medical Care Program; MCCS = Melbourne Collaborative Cohort Study; MEC = Multiethnic Cohort Study of Diet and Cancer; MMAS = Massachusetts Male Aging Study; NE RIA = non-extraction radioimmunoassay; NSHDC = Northern Sweden Health and Disease Cohort *(*androg = androgens; E_2_ = estradiol); PCPT = Prostate Cancer Prevention Trial; PHS *=* Physicians' Health Study; PLCO = Prostate, Lung, Colorectal and Ovarian Cancer Screening Trial; ProtecT = Prostate Testing for Cancer and Treatment; RBS = Rancho Bernardo Study; SHBG = Sex hormone binding globulin

†Intra-assay.

^a^ Inter-assay.

‡Intra-and inter-assay range.

^b^ Not specified.

All studies were either of a prospective cohort design[13–30] or were a prospective observational study within a randomized trial[31–36]. This analysis used secondary data, therefore ethical approval for this analysis was not necessary. However, each study individually obtained ethical approval. Details of ethical approval, participant consent and study design can be found in the original publications[13–36]. Participants were considered eligible for this analysis if they had information on at least one of the circulating sex hormones or SHBG concentrations, had not been diagnosed with prostate cancer by the time of censoring, were 25 years or over at blood collection, had complete data on age at blood collection, height, and weight, and were not known to be on androgen therapy at blood collection. Overall, these exclusion criteria resulted in 12,330 participants (out of a total of 14,092; S1 Fig) from 25 studies being included in these analyses (Table 2).

**Table 2:** Participant characteristics by study.

Study, country	N (% of total)	Mean age, years (SD)	Age range, years	Year of birth	Mean height, cm (SD)	Mean BMI, kg/m^2^ (SD)	% Current drinkers (median daily alcohol consumption, g alcohol)	% Current smokers (median number of daily cigarettes)	% White ethnic group	% Married/ cohabiting at blood collection	% University degree	% Family history of prostate cancer
ATBC, Finland	231 (1.9%)	60.5 (5.2)	50–69	1915–1936	173.8 (6.8)	25.9 (3.7)	85.4 (8.5)	100 (20)	-	82.3	5.6	2.5
BLSA, USA	112 (0.9%)	56.4 (12.8)	30–84	1895–1948	176.8 (6.0)	25.7 (3.0)	-	21.4	95.5	91.8	57.1	-
CARET, USA	300 (2.5%)	62.8 (6.0)	47–77	1913–1945	174.9 (7.6)	27.7 (4.4)	68.2 (5)	50.0 (20)	93.7	85.9	24.2	3.3
CHDS, USA	396 (2.4%)	34.9 (3.2)	25–50	1912–1941	178.7 (6.8)	24.7 (2.7)	77.5 (6)	50.6 (20)	63.1	99.7	34.5	-
EPIC, Europe	638 (5.2%)	60.9 (6.2)	43–76	1918–1952	172.6 (7.0)	27.0 (3.6)	87.1 (13)	27.8 (15)	100	89.1	22.9	-
EPIC Norfolk, Europe	707 (5.7%)	66.7 (6.5)	45–78	1918–1950	172.9 (6.4)	26.6 (3.2)	73.8 (8)	9.3	99.9	88.3	12.5	1.7
FMC, Finland	298 (2.4%)	57.9 (9.9)	33–80	1890–1937	169.6 (6.8)	26.1 (3.5)	-	34.2 (15)	100	85.2	-	-
HHS, Finland	291 (2.4%)	51.0 (3.8)	40–56	1924–1940	174.2 (6.2)	26.4 (3.1)	-	-	-	-	-	-
HPFS, USA	682 (5.5%)	65.1 (7.4)	46–80	1913–1946	178.1 (6.4)	26.0 (3.5)	73.5 (6)	3.9 (20)	99.4	93.0	100	10.3
JACC, Japan	97 (0.8%)	67.8 (5.8)	58–83	1904–1934	159.5 (7.1)	22.4 (2.7)	52.6 (2)	37.2 (20)	0	93.8	2.7	-
Janus, Norway	1,153 (9.4%)	46.4 (4.2)	33–63	1910–1947	176.2 (6.8)	25.1 (3.2)	-	62.5 (12)	100	-	-	-
JHCS 1988, Japanese in Hawaii	98 (0.8%)	63.4 (5.6)	52–74	1900–1919	162.2 (5.4)	23.1 (2.8)	74.5 (5.5)	36.7 (20)	0	90.8	8.2	-
JHCS 1996, Japanese in Hawaii	139 (1.1%)	61.9 (5.7)	52–74	1900–1919	162.8 (6.4)	23.2 (2.8)	78.4 (5)	28.1 (20)	0	94.2	10.1	-
JPHC, Japan	399 (3.2%)	59.1 (6.6)	41–71	1923–1949	162.0 (6.4)	23.3 (2.6)	71.2 (23)	40.6 (20)	0	91.0	-	-
KPMCP, USA	212 (1.7%)	71.8 (4.5)	60–85	1882–1906	169.9 (6.7)	25.8 (3.1)	69.7 (10)	17.9 (30)	98.6	82.7	5.4	-
MCCS, Australia	1,058 (8.6%)	58.3 (7.2)	40–72	1918–1954	172.2 (7.3)	27.2 (3.7)	78.3 (13)	13.0 (20)	100	81.0	22.2	-
MEC, USA	922 (7.5%)	68.7 (7.2)	48–84	1918–1948	174.2 (7.7)	27.1 (4.2)	90.9 (9)	12.6 (15)	13.2	78.1	32.2	9.0
MMAS, USA	651 (5.3%)	57.6 (7.0)	41–70	1916–1946	175.8 (7.2)	27.5 (4.5)	-	23.5	98.8	77.1	37.8	-
NSHDC E2, Sweden	465 (3.8%)	57.6 (4.0)	40–61	1927–1948	175.8 (5.9)	26.6 (3.5)	-	21.3	100	-	-	-
NSHDC androg, Sweden	382 (3.1%)	57.2 (4.5)	39–61	1927–1958	175.8 (6.2)	26.5 (3.4)	100.0 (4)	17.4	100	85.6	12.4	
PCPT, USA and Canada	1,025 (8.3%)	63.3 (5.5)	55–83	1911–1940	177.4 (7.0)	27.7 (4.0)	68.9 (3)	7.6 (20)	83.9	87.7	37.1	20.7
PHS, USA	376 (3.0%)	61.3 (7.5)	41–77	1905–1941	177.9 (7.0)	24.6 (2.5)	84.4 (5)	8.5 (20)	96.5	-	100	-
PLCO, USA	858 (7.0%)	64.8 (4.8)	54–75	1919–1944	177.9 (6.5)	27.4 (3.9)	70.0 (4)	9.1 (20)	100	86.9	42.2	6.1
ProtecT, UK	539 (4.4%)	61.5 (5.1)	50–70	1930–1949	175.2 (6.5)	26.6 (3.5)	84.9 (18)	10.5	-	-	-	4.1
RBS, USA	301 (2.4%)	69.4 (9.0)	47–85	1898–1938	174.4 (6.8)	26.0 (3.6)	68.8 (10)	7.3 (20)	100	90.4	22.6	-
Missing data (%)	-	-	-	-	-	-	31.1	12.2	9.0	26.3	27.3	55.5
Overall	12,330	59.9 (9.9)	25–85	1882–1958	174.4 (7.8)	26.4 (3.8)	77.1 (8)	23.7 (20)	89.4	86.5	35.8	8.2

Abbreviations: ATBC = The Alpha-Tocopherol, Beta-Carotene Cancer Prevention Study; BLSA = The Baltimore Longitudinal Study of Aging; BMI = body mass index; CARET = The Carotene and Retinol Efficacy Trial; CDHS = Child Health and Development Studies; EPIC = European Prospective Investigation into Cancer and Nutrition; FMC = Finnish Mobile Clinic Health Examination Survey; HHS = Helsinki Heart Study; HPFS = Health Professionals Follow-Up Study; JACC = Japan Collaborative Cohort Study; JPHC = Japan Public Health Center-based Prospective Study; JHCS = Japan-Hawaii Cancer Study; KPMCP = Kaiser Permanente Medical Care Program; MCCS = Melbourne Collaborative Cohort Study; MEC = Multiethnic Cohort; MMAS = Massachusetts Male Aging Study; NSHDC = Northern Sweden Health and Disease Cohort (androg *=* androgens; E_2_ = estradiol); PCPT = Prostate Cancer Prevention Trial; PHS *=* Physicians' Health Study; PLCO Prostate, Lung, Colorectal and Ovarian Cancer Screening Trial; ProtecT = Prostate Testing for Cancer and Treatment; RBS = Ranch Bernardo Study; SD = standard deviation

Free testosterone and free estradiol concentrations were calculated using the law of mass action[37, 38] based on recorded SHBG, testosterone and estradiol values and assuming a constant albumin concentration of 43 g/L.

### Statistical analysis

Hormone and SHBG concentrations were logarithmically transformed to approximate normal distributions. Categories investigated were selected *a priori* and cut-points chosen based on sample size and the data distribution. The analyses examined associations of hormone concentrations with age (25–49 [mean age = 43.5], 50–54, 55–59, 60–64, 65–69, 70–74, 75+ years), height (<160.0, 160.0–164.9, 165.0–169.9, 170.0–174.9, 175.0–179.9, 180.0–184.9, 185.0–189.9, 190.0+ cm), BMI (<20.0, 20.0–22.4, 22.5–24.9, 25.0–27.4, 27.5–29.9, 30.0–32.4, 32.5–34.9, 35.0–37.4, 37.5+ kg/m^2^), waist circumference (<90.0, 90.0–94.9, 95.0–99.9, 100.0–104.9, 105.0+ cm) and waist-to-hip ratio (<0.900, 0.900–0.933, 0.934–0.966, 0.967–0.999, 1.000+). Health behaviours investigated included smoking status (never, former, current: <15, 15–29, 30+ cigarettes per day) and alcohol consumption (none, 1–9, 10–19, 20–29, 30–39, 40–49, 50–59, 60–69, 70+ g alcohol per day). Ethnic/racial group was categorised as white, African American/Caribbean, Hispanic/Latino, East Asian, and other ethnic/racial group. Other possible determinants that were examined included: time of day of blood collection (before 09:00, 09:00–11:59, 12:00–14:59, 15:00 onwards); educational status (no secondary/high school education, secondary school, college and university); marital status (currently married/cohabiting, not currently married/cohabiting) and family history of prostate cancer (no, yes: defined as a father and/or brother diagnosed with prostate cancer).

Partial correlations between the sex hormones and SHBG were calculated using study-specific standardized values: (*x*_*jk*_*-m*_*j*_)/*s*_*j*_, where *m*_*j*_ and *s*_*j*_ denote the mean and standard deviation of the log-transformed hormone concentrations in study *j* and *x*_*jk*_ is an observation from that study. These standardized values were adjusted for age at blood collection and BMI (included as categorical variables, described above). Geometric mean concentrations of sex hormones and SHBG were calculated using predicted values from analysis of variance models scaled to the overall geometric mean concentration and adjusted for study, age at blood collection, and BMI (with the exception of when we analysed the associations of age and BMI with hormone concentrations, when these variables were not included as adjustment covariates). Analyses of smoking and alcohol consumption were mutually adjusted for each other. To investigate the relationship of hormone concentrations with ethnicity/race, studies were limited to the three studies that had sufficient representation from men across multiple ethnic/racial groups (Child Health and Development Studies, the Multi-Ethnic Cohort and the Prostate Cancer Prevention Trial). Heterogeneity of means by category of each characteristic was tested using the F test. Where appropriate, a test for trend was calculated using the analysis of variance test, with the categorical variables entered as linear values scored consecutively as 1, 2, 3 etc. Owing to the highly skewed distribution of alcohol consumption, the test for trend was calculated based on median values within each category. To test for trend by smoking status, never and former smokers were combined and coded as 0; light, medium and heavy smokers were coded as 1, 2 and 3, respectively.

Most variables had a small number of missing values (Table 2). Time of day at blood collection was missing for 44.8% of men and was therefore not included as a covariate in the analyses. To enable adjustment for study, each study had to contain observations in a minimum of two categories for each primary exposure to be included in the respective exposure analysis. Assay methods for each hormone varied between studies (Table 1); some studies used “extraction” assays to measure circulating sex hormones (immunoassays preceded by extraction into an organic solvent and celite column chromatography), which generally provide higher sensitivity and specificity than non-extraction or “direct” assays. Heterogeneity between studies and by assay method was tested using a study-by-factor and assay-by-factor interaction term (fitted separately) in the analysis of variance, and assessed using the F test.

All statistical tests were two-sided and due to the multiple tests the statistical significance threshold was p<0.01. Data analysis was carried out using Stata Statistical Software release 14.1 (Stata Corp., College Station, TX, USA).

### Sensitivity analyses

To examine effect modification by these factors, separate analyses were performed stratified by: age (<55, 55–69, 70+ years); BMI (<20.0, 20.0–29.9, 30.0+ kg/m^2^); and time of day (morning and afternoon), in comparison to a restricted dataset. The analyses were also repeated after restricting the dataset to: i) white men to examine whether ethnicity/race was a confounder, ii) men with hormone concentrations that were within the range of [lower quartile– 3*interquartile range, upper quartile + 3*interquartile range] of each respective study to examine the effect of removing outliers (n = 63).

## Results

25 studies contributed to the analysis, including 12,330 participants (Table 2). Participants were born between 1882–1958 and age at blood collection ranged from 25–85 years (mean = 59.9 years; standard deviation = 9.9 years). The men were predominantly white (89.4%).

### Correlations between hormones

After taking into account age, BMI and study, most sex hormones were positively correlated with each other (Table 3). Testosterone was most strongly correlated with free testosterone (r = 0.77), DHT (r = 0.54) and SHBG (r = 0.52). DHEAS was correlated with androstenedione (r = 0.40) and A-diol-G (r = 0.23). Estradiol was strongly correlated with free estradiol (r = 0.88) and estrone (r = 0.54), and weakly correlated with SHBG (r = 0.10).

**Table 3:** Partial correlation coefficients in control subjects between log-transformed concentrations of sex hormones and SHBG.

	Androstenedione	A-diol-G	DHEAS	Testosterone	Free testosterone	DHT	Estradiol	Free estradiol	Estrone	SHBG
Androstenedione	1									
A-diol-G	0.23*	1								
DHEAS	0.40*	0.21*	1							
Testosterone	0.29*	0.25*	0.07*	1						
Free testosterone	0.29*	0.28*	0.15*	0.77*	1					
DHT	0.20*	0.22*	0.03	0.54*	0.34*	1				
Estradiol	0.13*	0.14*	0.06*	0.33*	0.34*	0.19*	1			
Free estradiol	0.09*	0.13*	0.11*	0.05*	0.37*	-0.03	0.88*	1		
Estrone	0.45*	0.18*	0.15	0.27*	0.26*	0.19*	0.54*	0.50*	1	
SHBG	0.10*	0.04*	-0.08*	0.52*	-0.11*	0.36*	0.10*	-0.36*	0.09*	1

Measurements are standardised by study and adjusted by age and BMI.

*P<0.01.

Abbreviations: A-diol-G = Androstanediol glucuronide; DHEAS = Dehydroepiandrosterone sulfate; DHT = Dihydrotestosterone; SHBG = sex hormone-binding globulin.

### Age at blood collection

**Fig 1:**
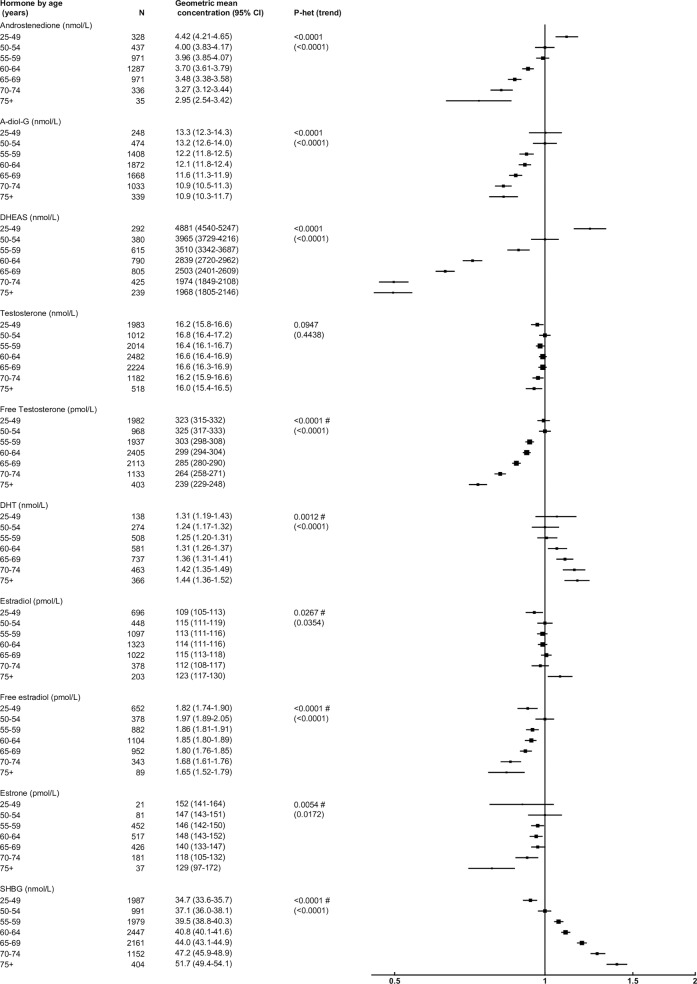
Relative geometric mean concentrations* of male sex hormones by age category. *relative to 50–54 years, adjusted for study and BMI. #significant interaction with study P<0.01. Abbreviations: A-diol-G = Androstanediol glucuronide; CI = confidence interval; DHEAS = Dehydroepiandrosterone sulfate; DHT = Dihydrotestosterone; SHBG = sex hormone-binding globulin.

After adjusting for study and BMI, age was associated with concentrations of all hormones, except testosterone and estradiol (Fig 1). Compared to men aged 50–54 years, those aged 75+ years had circulating concentrations of DHEAS, free testosterone, androstenedione, A-diol-G and free estradiol that were 50%, 27%, 26%, 17%, 16% lower, respectively. In contrast, SHBG and DHT concentrations were 39% and 16% higher, respectively. There was significant heterogeneity (P<0.01) between studies in the associations of free testosterone, DHT, estradiol, free estradiol, estrone and SHBG.

Although there was no evidence of a linear association between age and testosterone concentration, there was evidence of heterogeneity in the association of testosterone concentration with age by time of day of blood collection (F = 3.00; P = 0.0005). For men whose blood was collected in the morning, younger men (aged 50–54 years) had 10% higher circulating concentrations of total testosterone than those aged 75+ years, while age was not associated with total testosterone concentration in men whose blood was collected in the afternoon (S2 Fig).

### BMI

**Fig 2:**
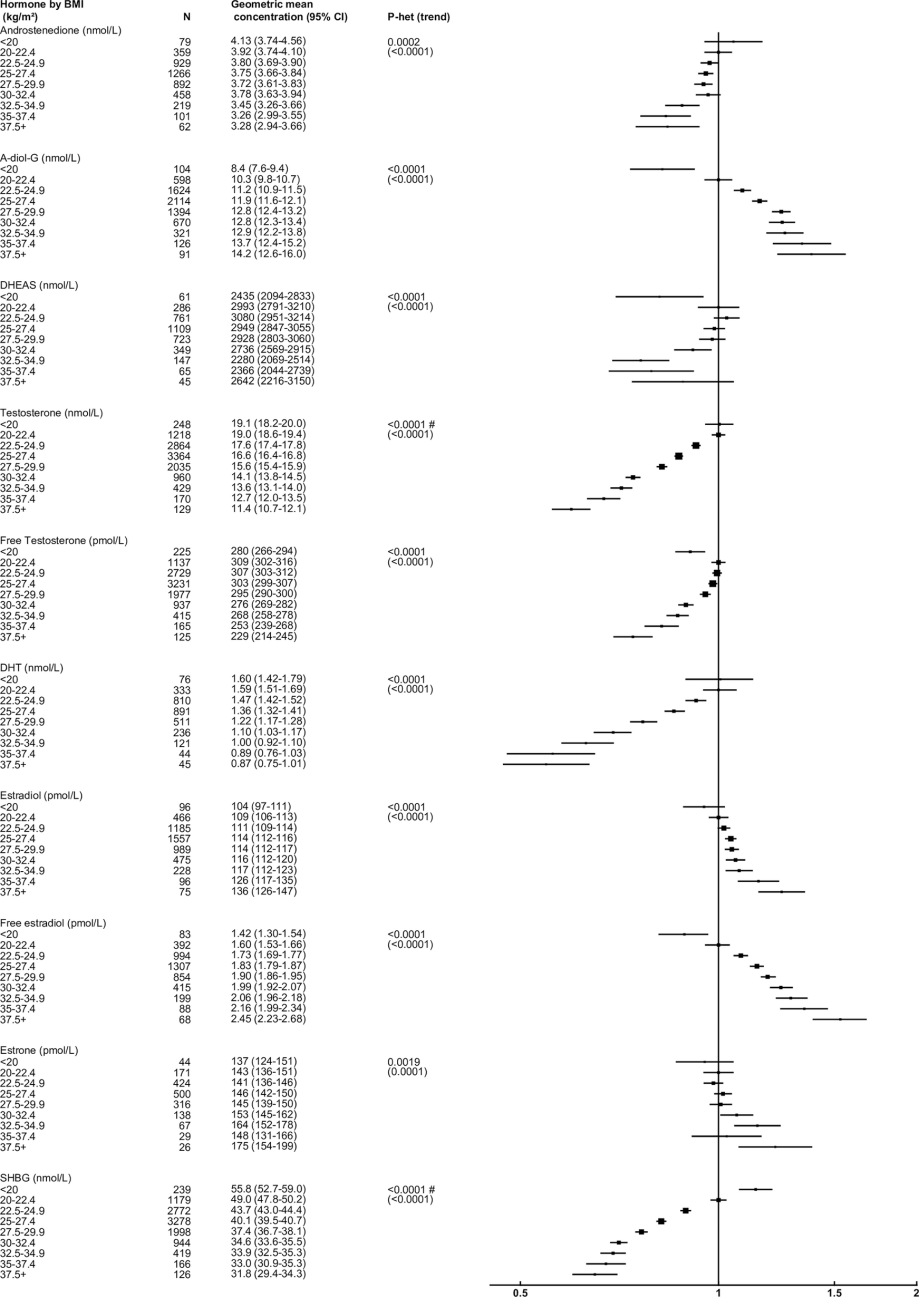
Relative geometric mean concentrations* of male sex hormones by BMI category. *relative to BMI 20.0–22.4 kg/m^2^, adjusted for study and age. #significant interaction with study P<0.01. Abbreviations: A-diol-G = Androstanediol glucuronide; CI = confidence interval; DHEAS = Dehydroepiandrosterone sulfate; DHT = Dihydrotestosterone; SHBG = sex hormone-binding globulin.

After adjusting for study and age, BMI was strongly associated with all sex hormones and SHBG (Fig 2). Compared to men with a BMI 20.0–22.4 kg/m^2^, those with a BMI ≥37.5 kg/m^2^ had concentrations of DHT, testosterone, SHBG, free testosterone, androstenedione and DHEAS that were 45%, 40%, 35%, 26%, 16% and 12% lower, respectively. In contrast, concentrations of free estradiol, A-diol-G, estradiol and estrone were 53%, 38%, 25% and 22% higher, respectively. These associations remained broadly similar when using waist circumference or WHR (S3 and S4 Figs), although when data were divided into fifths the magnitudes of the associations were smaller with WHR than with BMI or waist circumference (data not shown). Differences in geometric mean hormone concentrations by waist circumference and WHR were attenuated following additional adjustment for BMI, although testosterone and SHBG remained significantly associated with both waist circumference and WHR (S5 and S6 Figs).

### Height

**Fig 3:**
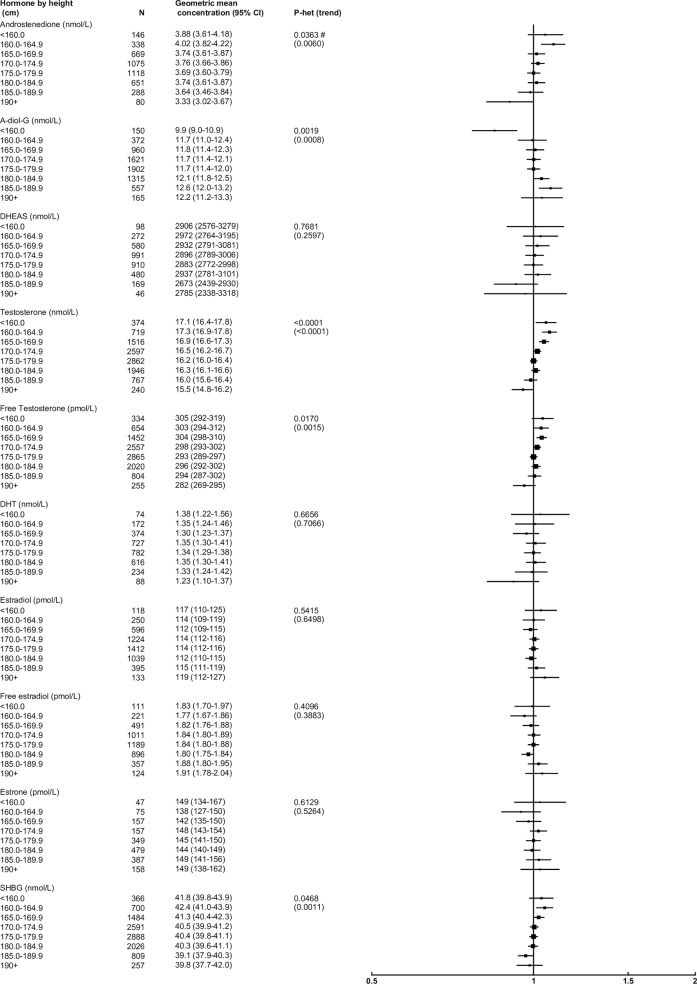
Relative geometric mean concentrations* of male sex hormones by height categories. *relative to 175.0–179.9 cm, adjusted for study, age and BMI. Abbreviations: A-diol-G = Androstanediol glucuronide; CI = confidence interval; DHEAS = Dehydroepiandrosterone sulfate; DHT = Dihydrotestosterone; SHBG = sex hormone-binding globulin.

After adjusting for study, age and BMI, height was associated with concentrations of several androgens and SHBG, but not estrogens (Fig 3). Compared to men with a height of 175–179 cm, those in the tallest category (≥190 cm) had concentrations of androstenedione, testosterone, free testosterone and SHBG that were 10%, 4%, 4%, and 2% lower, respectively, whilst A-diol-G concentration was 4% higher.

### Smoking

**Fig 4:**
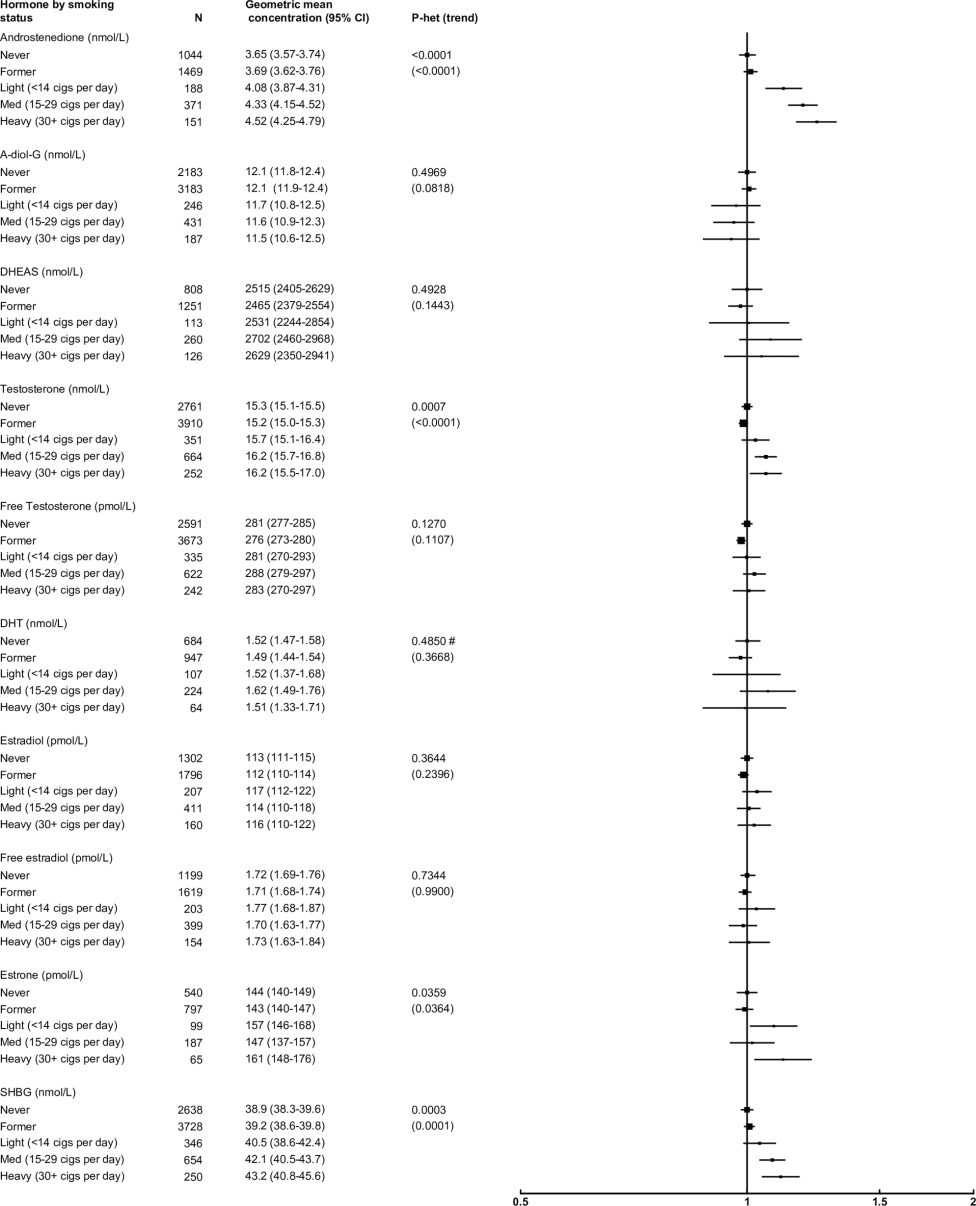
Relative geometric mean concentrations* of male sex hormones by smoking status. *relative to never smokers, adjusted for study, age, BMI and alcohol consumption. #significant interaction with study P<0.01. Abbreviations: A-diol-G = Androstanediol glucuronide; CI = confidence interval; DHEAS = Dehydroepiandrosterone sulfate; DHT = Dihydrotestosterone; SHBG = sex hormone-binding globulin.

After adjusting for study, age, BMI and alcohol consumption, smoking status was positively associated with concentrations of androgens and SHBG, but not estrogens (Fig 4). Compared with never smokers, heavy smokers (30+ cigarettes/day) had concentrations of androstenedione, SHBG and testosterone that were 24%, 11% and 6% higher, respectively.

### Alcohol

**Fig 5:**
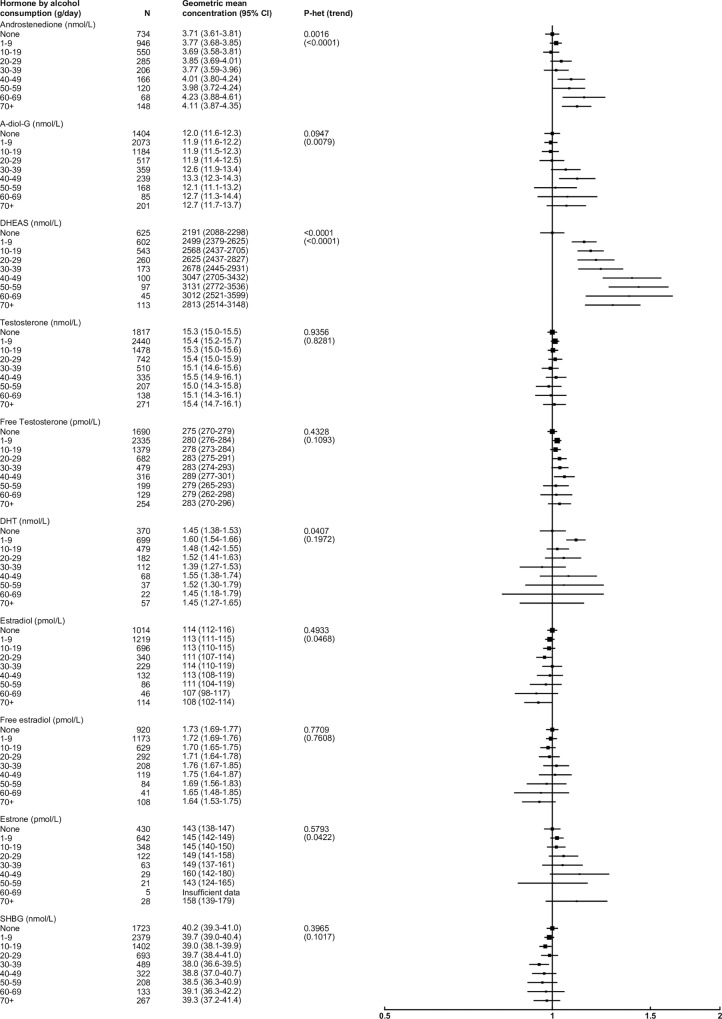
Relative geometric mean concentrations* of male sex hormones by alcohol consumption categories. *relative to non-drinkers, adjusted for study, age, BMI and smoking status. Abbreviations: A-diol-G = Androstanediol glucuronide; CI = confidence interval; DHEAS = Dehydroepiandrosterone sulfate; DHT = Dihydrotestosterone; SHBG = sex hormone-binding globulin.

After adjusting for study, age, BMI and smoking, alcohol consumption was associated with higher concentrations of several androgens, but was not associated with estrogens (Fig 5). In particular, compared with non-drinkers, those who drank ≥70 g alcohol/day had a 28% higher concentration of DHEAS. High alcohol consumption was also moderately associated with higher concentrations of androstenedione and A-diol-G (11% and 6% higher, respectively). Restricting the analysis to non-smokers (n = 6,953) did not materially alter these results.

### Ethnic/racial group

**Fig 6:**
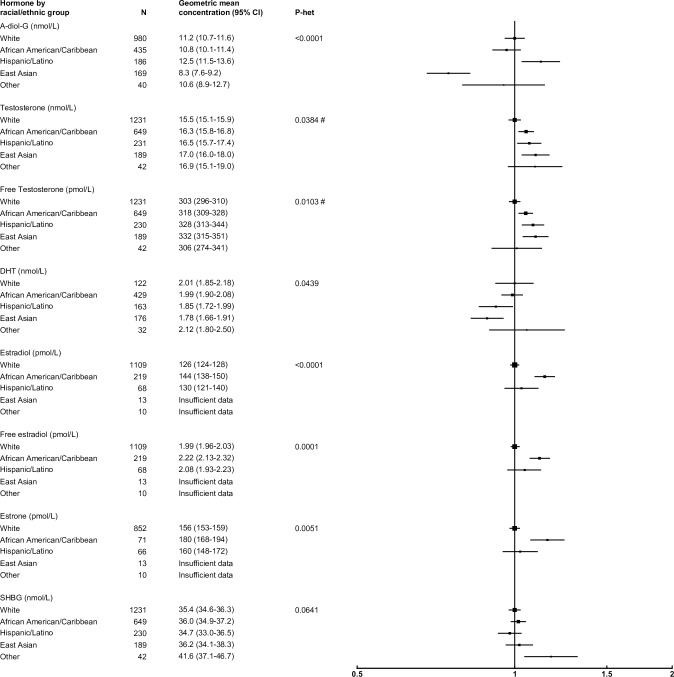
Relative geometric mean concentrations* of male sex hormones by ethnic/racial group. *relative to whites, adjusted for study, age and BMI. #significant interaction with study P<0.01. Abbreviations: A-diol-G = Androstanediol glucuronide; CI = confidence interval; DHEAS = Dehydroepiandrosterone sulfate; DHT = Dihydrotestosterone; SHBG = sex hormone-binding globulin.

After adjusting for study, age and BMI, most sex hormones did not differ by ethnic/racial group, with the exception of A-diol-G and estrogens (Fig 6). Compared with white men, A-diol-G concentration was 25% lower in East Asian men and 12% higher in Hispanic/Latino men, while estrone, estradiol and free estradiol concentrations were 15%, 14% and 12% higher in African American men than whites.

### Time of blood collection

**Fig 7:**
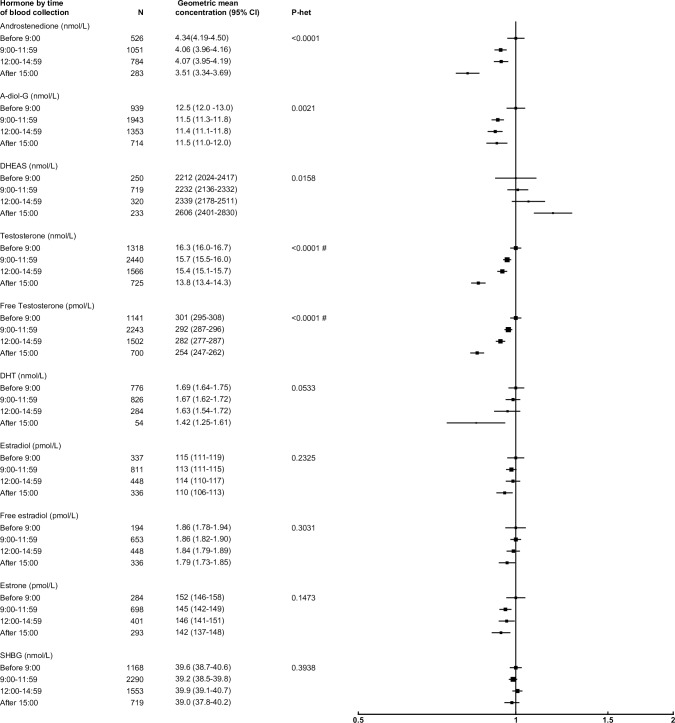
Relative geometric mean concentrations* of male sex hormones by time of blood collection. *relative to before 09:00, adjusted for study, age and BMI. #significant interaction with study P<0.01. Abbreviations: A-diol-G = Androstanediol glucuronide; CI = confidence interval; DHEAS = Dehydroepiandrosterone sulfate; DHT = Dihydrotestosterone; SHBG = sex hormone-binding globulin.

After adjusting for study, age and BMI, several sex hormones were associated with time of blood collection (Fig 7). Compared with men whose blood was collected before 09:00, participants whose blood was collected at 15:00 or later had concentrations of androstenedione, testosterone, free testosterone and A-diol-G that were 19%, 16%, 15% and 8% lower, respectively.

### Sociodemographic factors and health

After adjusting for study, age and BMI several sex hormones had small associations with sociodemographic factors. In comparison to men with no secondary/high school qualifications, men with a university education had concentrations of SHBG, androstenedione and testosterone and that were 7%, 6% and 5% lower, respectively (S7 Fig). In comparison to men who were married/cohabiting, men who were not married/cohabiting had concentrations of estrone, androstenedione, free estradiol and estradiol and that were 12%, 7%, 6% and 6% higher, respectively (S8 Fig). Hormone concentrations were not associated with a family history of prostate cancer (S9 Fig).

### Heterogeneity by study and assay

Some heterogeneity between studies in the associations of sex hormone concentrations with anthropometric and sociodemographic factors was observed (see Figs 1–7). Much of the observed heterogeneity was due to differences in the magnitude rather than the direction of associations (data not shown). Some of the heterogeneity between studies may have been caused by differences in assay type. There was heterogeneity by assay type in the associations of DHT and estradiol concentrations with age. There was no increase in DHT with age in studies that used assays without an extraction step prior to assay, whilst age was associated with higher DHT concentration in studies with an extraction step. For estradiol, the positive association with age was stronger in studies that used an extraction step than in those that did not.

### Sensitivity analyses

Stratification by age, BMI and time of day did not materially affect the results (data not shown), with the exception of the association of total testosterone with age (see above). The results also remained broadly unchanged after restricting the dataset to white men and excluding within study hormone outliers (data not shown).

## Discussion

This international collaboration has brought together and analysed data from over 12,300 men on the associations of various anthropometric, behavioural and sociodemographic factors with circulating sex hormone and SHBG concentrations. Our findings suggest that age, body composition, and to a lesser extent smoking status and alcohol consumption, may be important determinants of circulating sex hormone concentrations.

**Fig 8:**
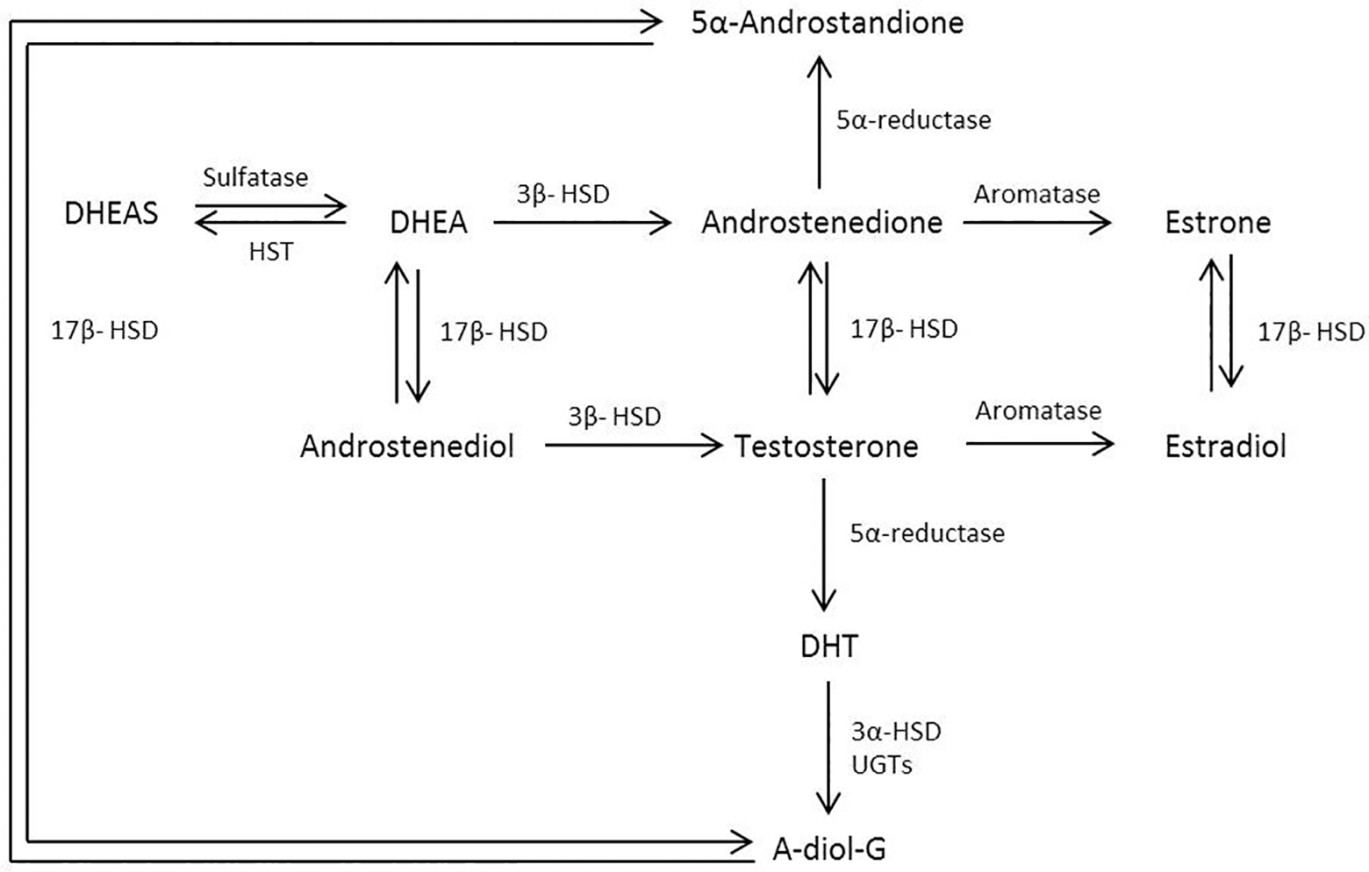
Simplified schematic of the biosynthesis pathway of sex hormones. Abbreviations: A-diol-G = Androstanediol glucuronide; DHEAS = Dehydroepiandrosterone sulfate; DHT = Dihydrotestosterone; HSD = Hydroxysteroid dehydrogenase; HST = Hydroxysteroid sulfotransferase; UGT = Uridine 5'-diphospho-glucuronosyltransferase.

As expected, hormones were strongly correlated with other hormones close to them on the sex steroid pathway (Fig 8). Testosterone is primarily secreted by the testes. The adrenal glands are another source of androgens, including DHEAS and androstenedione, both of which can ultimately be converted to testosterone and/or DHT in peripheral tissues. Androgens can also be converted into estrogens, primarily in the testes and adipose tissue. <3% of circulating testosterone and estradiol is bioavailable to the tissues, or “free”, the remainder is primarily bound to either SHBG or albumin[39].

Age was strongly associated with circulating SHBG and androgens, with the exception of total testosterone concentration. Other studies have generally found that total testosterone declines with age[12, 40–44], although more recent research suggests that the observed decline may be partly due to confounding by obesity and comorbidities[45–49]. Our analyses found no significant overall association of total testosterone with age. However, stratification by time of day showed a significant linear decline with age only in men whose blood was collected in the morning; circulating testosterone concentrations are highest in the morning and decline throughout the day. However, this diurnal rhythm is blunted as men age[50, 51]. The marked positive association of SHBG concentration with age may be related to a lower insulin-like growth factor-1 concentration in older individuals[52]. Adrenal sensitivity to adrenocorticotrophic hormone has also been shown to decline with age[53], which results in a substantially reduced production of DHEAS and, to a lesser extent, androstenedione[54, 55]. Despite this age-associated decline in androstenedione, DHT concentration was higher in older men, which might be due to the reduced conversion of DHT to A-diol-G[56].

BMI was strongly associated with concentrations of all the sex hormones and SHBG. A higher BMI was associated with markedly lower SHBG concentration, which may be due to high insulin[57]. Some recent evidence indicates that this BMI-SHBG association may involve low grade inflammation and/or increased liver fat[58, 59]. The reduction in SHBG may lead to a fall in testosterone production via a negative feedback loop. Increasing adipose tissue also leads to higher aromatase activity[60], which converts androgens to estrogens, leading to higher concentrations of circulating estrogens. This, together with the low concentrations of SHBG, results in markedly higher concentrations of free estradiol in obese men. Our finding of a strong linear association of A-diol-G concentration with BMI suggests an increase in peripheral androgen metabolism with greater adiposity. Obesity leads to dysregulation of the hypothalamic-pituitary-adrenal axis[57, 61] and may therefore result in lower DHEAS and androstenedione production. After adjustment for BMI, waist circumference and WHR each remained significantly associated with testosterone and SHBG concentrations. This suggests that visceral fat, or other factors correlated with waist circumference and WHR, may be important predictors of androgen and SHBG concentrations[62].

Testosterone, free testosterone and SHBG were weakly inversely associated with height, and A-diol-G was positively associated with height. While estrogens are involved in the control of growth in height[63] and affect growth hormone secretion[64], there is some evidence to suggest that androgens may also be involved[65]. However, it is unclear to what extent circulating sex hormone concentrations during childhood and adolescence correlate with concentrations later in life.

The mechanisms through which tobacco may affect hormone concentrations are complex because cigarette smoke contains a wide range of endocrine disruptors, some of which exert opposing physiological effects[66, 67]. Smoking was associated with higher concentrations of testosterone and SHBG, which resulted in a relatively unchanged free testosterone concentration[68–70]. In our analysis smoking was also associated with elevated circulating androstenedione concentration.

Alcohol consumption was associated with moderately higher androgen concentrations, particularly for DHEAS, as observed previously[71–73], and to a lesser extent with androstenedione, possibly involving increased adrenal secretion and inhibition of hydroxysteroid dehydrogenase[73]. The large association with DHEAS may be related to the long half-life of this metabolite. Alcohol consumption was also moderately positively associated with A-diol-G, although findings from other studies are inconsistent[74, 75]. While chronic excessive alcohol consumption is a well-known cause of low testosterone in men[76], we did not find an inverse association of alcohol with testosterone concentration in the current analyses, perhaps because the levels of alcohol intake were not sufficient to induce a detrimental biological effect.

Three studies provided data from men across several ethnic/racial groups and we found that ethnic/racial group was significantly associated with circulating A-diol-G concentration, which was lower in East Asian men compared to all other ethnic/racial groups, as observed previously[77–79]. Estrone, estradiol and free estradiol concentration were also significantly higher in African Americans, as reported elsewhere[80], although the mechanism for this remains unclear. Further inference regarding the association between ethnicity/race and other hormone concentrations was not possible due to the relatively low representation of non-white men.

Heterogeneity between the included studies was present for several associations. Further investigation showed that this was generally caused by differences in the magnitude of association rather than the direction. Heterogeneity may have been caused by differences in sample populations, study design, and for testosterone distribution of time of day of blood draw. Additionally, some of the observed heterogeneity may have been attributable to differences in assay type. In this analysis, no study used mass spectrometry, often considered the gold standard method of measurement for sex hormones[81]. Instead, the majority of sex hormones were measured using extraction or non-extraction immunoassays. Non-extraction assays generally have a lower sensitivity and specificity for hormone concentrations than extraction assays, and differences in assay performance may explain some of the differences observed in hormone concentrations (Table 1).

The cross-sectional nature of this analysis means that it was not possible to determine whether the associations found here are causal or due to reverse causation. Furthermore, younger age groups are under-represented. Data regarding other potential confounders such as physical activity and dietary factors were not collected in this centralized dataset, although only weak associations with hormone concentrations have been reported[82, 83], therefore any related confounding of the studied associations is likely to be small.

In summary, this analysis of individual participant data from 25 studies shows that circulating sex hormones in men are strongly associated with age and BMI, and to a lesser extent, with smoking status and alcohol consumption. These associations may enable a greater understanding of how risk factors are associated with the etiology of hormone-related diseases.

## Supporting information

S1 Table Individual study contact details. Abbreviations: ATBC = The Alpha-Tocopherol, Beta-Carotene Cancer Prevention Study; BLSA = The Baltimore Longitudinal Study of Aging; CARET = The Carotene and Retinol Efficacy Trial; CDHS = Child Health and Development Studies; EPIC = European Prospective Investigation into Cancer and Nutrition; FMC = Finnish Mobile Clinic Health Examination Survey; HHS = Helsinki Heart Study; HPFS = Health Professionals Follow-Up Study; JACC = Japan Collaborative Cohort Study; JPHC = Japan Public Health Center-based Prospective Study; JHCS = Japan-Hawaii Cancer Study; KPMCP = Kaiser Permanente Medical Care Program; MCCS = Melbourne Collaborative Cohort Study; MEC = Multiethnic Cohort; MMAS = Massachusetts Male Aging Study; NSHDC = Northern Sweden Health and Disease Cohort; PCPT = Prostate Cancer Prevention Trial; PHS *=* Physicians' Health Study; PLCO Prostate, Lung, Colorectal and Ovarian Cancer Screening Trial; ProtecT = Prostate Testing for Cancer and Treatment; RBS = Ranch Bernardo Study.
(DOCX)

S1 Fig Participant selection chart. (PNG)

S2 Fig Relative geometric mean concentrations* of testosterone concentration by age, stratified by time of blood collection (morning or afternoon). *Relative to overall geometric mean of testosterone, adjusted for study and BMI. Test of heterogeneity between morning and afternoon blood collection: F = 3.00; P = 0.0005. Abbreviations: CI = confidence interval.
(EMF)

S3 Fig Relative geometric mean concentrations* of male sex hormones by waist circumference (cm). *relative to 90.0–94.9 cm, adjusted for study and age. Abbreviations: A-diol-G = Androstanediol glucuronide; CI = confidence interval; DHEAS = Dehydroepiandrosterone sulfate; DHT = Dihydrotestosterone; SHBG = sex hormone-binding globulin.
(EMF)

S4 Fig Relative geometric mean concentrations* of male sex hormones by waist-to-hip ratio. *relative to 0.90–0.93, adjusted for study and age. Abbreviations: A-diol-G = Androstanediol glucuronide; CI = confidence interval; DHEAS = Dehydroepiandrosterone sulfate; DHT = Dihydrotestosterone; SHBG = sex hormone-binding globulin.
(EMF)

S5 Fig Relative geometric mean concentrations* of male sex hormones by waist circumference (cm). *relative to 90.0–94.9 cm, adjusted for study, age and BMI. Abbreviations: A-diol-G = Androstanediol glucuronide; CI = confidence interval; DHEAS = Dehydroepiandrosterone sulfate; DHT = Dihydrotestosterone; SHBG = sex hormone-binding globulin.
(EMF)

S6 Fig Relative geometric mean concentrations* of male sex hormones by waist-to-hip ratio. *relative to 0.90–0.93, adjusted for study, age and BMI. Abbreviations: A-diol-G = Androstanediol glucuronide; CI = confidence interval; DHEAS = Dehydroepiandrosterone sulfate; DHT = Dihydrotestosterone; SHBG = sex hormone-binding globulin.
(EMF)

S7 Fig Relative geometric mean concentrations* of male sex hormones by education status. *relative to < secondary/high school, adjusted for study, age and BMI. Abbreviations: A-diol-g = Androstanediol glucuronide; CI = confidence interval; DHEAS = Dehydroepiandrosterone sulfate; DHT = dihydrotestosterone; SHBG = sex hormone-binding globulin.
(EMF)

S8 Fig Relative geometric mean concentrations* of male sex hormones by marriage status. *relative to married/cohabiting, adjusted for study, age and BMI. # significant interaction with study P<0.01. Abbreviations: A-diol-G = Androstanediol glucuronide; CI = confidence interval; DHEAS = Dehydroepiandrosterone sulfate; DHT = Dihydrotestosterone; SHBG = sex hormone-binding globulin.
(EMF)

S9 Fig Relative geometric mean concentrations* of male sex hormones by family history of prostate cancer. *relative to no family history of prostate cancer, adjusted for study, age and BMI. Abbreviations: A-diol-G = Androstanediol glucuronide; CI = confidence interval; DHEAS = Dehydroepiandrosterone sulfate; DHT = Dihydrotestosterone; SHBG = sex hormone-binding globulin.
(EMF)
