# Supplementary material for: Circulating sex hormones in relation to anthropometric, sociodemographic and behavioural factors in an international dataset of 12,300 men
Source: PLoS One. 2017 Dec 27;12(12):e0187741. doi: 10.1371/journal.pone.0187741 (PMC5744924; doi:10.1371/journal.pone.0187741)
Supplement: S1 Table — Abbreviations: ATBC = The Alpha-Tocopherol, Beta-Carotene Cancer Prevention Study; BLSA = The Baltimore Longitudinal Study of Aging; CARET = The Carotene and Retinol Efficacy Trial; CDHS = Child Health and Development Studies; EPIC = European Prospective Investigation into Cancer and Nutrition; FMC = Finnish Mobile Clinic Health Examination Survey; HHS = Helsinki Heart Study; HPFS = Health Professionals Follow-Up Study; JACC = Japan Collaborative Cohort Study; JPHC = Japan Public Health Center-based Prospective Study; JHCS = Japan-Hawaii Cancer Study; KPMCP = Kaiser Permanente Medical Care Program; MCCS = Melbourne Collaborative Cohort Study; MEC = Multiethnic Cohort; MMAS = Massachusetts Male Aging Study; NSHDC = Northern Sweden Health and Disease Cohort; PCPT = Prostate Cancer Prevention Trial; PHS = Physicians' Health Study; PLCO Prostate, Lung, Colorectal and Ovarian Cancer Screening Trial; ProtecT = Prostate Testing for Cancer and Treatment; RBS = Ranch Bernardo Study. (DOCX) [file pone.0187741.s001.docx]

| Study |  |
| --- | --- |
| ATBC | The two international partners of this project - the National Cancer Institute (NCI) and the National Institute for Health and Welfare of Finland (THL) - have primary responsibility for project research management, coordination, and oversight. The Metabolic Epidemiology Branch (MEB) represents the NCI in this regard. Study proposals for collaboration are submitted to the NCI or NPHI Principal Investigators (Dr. Albanes (daa@nih.gov) and Dr. Männistö (satu.mannisto@thl.fi), respectively), and are reviewed at both institutions. |
| BLSA | Available upon request from the study website [https://www.blsa.nih.gov]. All requests submitted through the website are routed to the BLSA Data Sharing Proposal Review Committee that oversee all data requests/releases and the committee meets bi-monthly. |
| CARET | Available upon request from the study website [http://www.compass.fhcrc.org/caretWeb/requests/requestingInfo.aspx]. |
| CHDS | Available upon request from the study website [http://www.chdstudies.org/]. |
| EPIC | For information on how to submit an application for gaining access to EPIC data and/or biospecimens, please follow the instructions at [http://epic.iarc.fr/access/index.php]. |
| FMC | The Finnish Mobile Clinic Health Surveys welcome applications from and collaboration with research groups interested in carrying out epidemiological studies based on the FMC, FMCF or MFS data. A steering committee and the Biobank at the National Institute for Health and Welfare (THL) are responsible for the scientific evaluation and approval of applications. Use of the data is subject to strict ethical and legal regulations. All studies using those data have to be approved by regional committees responsible for medical and health research ethics. Delivery of data or biological samples outside Finland requires a transfer agreement to be signed by THL and the applicant institution. Further information about the surveys is available at [www.thl.fi/finnish-mobile-clinic]. |
| HHS | The Helsinki Health Study cohort offers wide opportunities for further longitudinal and comparative analyses across various domains of health and well-being in the social and work context. The data are kept at the Hjelt Institute, Department of Public Health, University of Helsinki, Finland. The team welcomes research proposals and initiatives. Collaboration is promoted and interested researchers and teams are encouraged to contact the Helsinki Health Study group, head Eero Lahelma (e-mail: eero.lahelma@helsinki.fi). Further information can be found at the study website [www.hjelt.helsinki.fi/]. |
| HPFS | Available upon request from the study website [https://content.sph.harvard.edu/hpfs/]. |
| JACC | See study website [http://publichealth.med.hokudai.ac.jp/jacc/] |
| Janus | Procedures and application forms can be found at: [http://www.kreftregisteret.no/en/Research/Janus-Serum-Bank/Project-handling/How-to-apply-for-serum-samples/]. |
| JHCS | See study website [http://www.kuakini.org/wps/portal/kuakini-research/research-home/for-researchers/apply] |
| JPHC | The data are owned by the Center for Public Health Sciences at the National Cancer Center, Japan. Researchers who have an interest in the analysis using JPHC data, please contact Dr. Shoichiro Tsugane, director, Center for Public Health Sciences, National Cancer Center, Japan, stsugane@ncc.go.jp. |
| KPMCP | Available upon request from the study website [https://rcp.kaiserpermanente.org/]. |
| MCCS | MCCS data are publicly available at [http://www.cancervic.org.au/research/epidemiology/health_2020]. The data are available to other researchers for approved collaborative research. |
| MEC | The MEC is committed to sharing data and biospecimens with qualified researchers to further our understanding of the determinants of cancer and other outcomes in diverse racial/ethnic populations. For access, a research proposal application will be required. Proposals are reviewed quarterly by the MEC Research Committee. To request the proposal template please contact Gail Ichida, gichida@cc.hawaii.edu |
| MMAS | Enquiries to Dr Varant Kupelian [varant.kupelian@gmail.com] |
| NSHDC | Available upon request from the study website [http://www.biobank.umu.se/biobank/biobank---for-researchers/access/]. |
| PCPT | See [http://www.swogstat.org/pcpthome.htm] for contact details |
| PHS | See [http://phs.bwh.harvard.edu/index.html] for contact details |
| PLCO | Available upon request from the study website [ https://biometry.nci.nih.gov/cdas/]. |
| ProtecT | Available upon request from the study website [http://www.bristol.ac.uk/population-health-sciences/projects/protect/about]. |
| RBS | Available upon request from the study website [https://knit.ucsd.edu/ranchobernardostudy/] |
