# Supplementary figures and images for: Circulating sex hormones in relation to anthropometric, sociodemographic and behavioural factors in an international dataset of 12,300 men
Source: PLoS One. 2017 Dec 27;12(12):e0187741. doi: 10.1371/journal.pone.0187741 (PMC5744924; doi:10.1371/journal.pone.0187741)

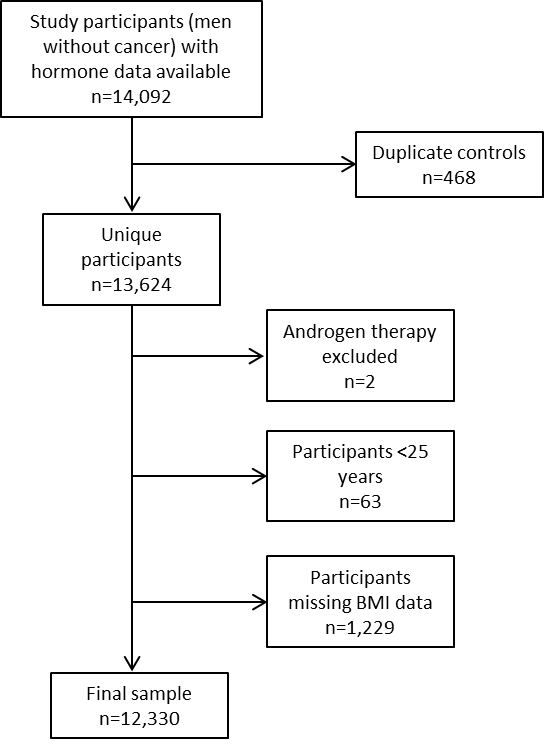

Supplement: S1 Fig — (PNG) [file pone.0187741.s002.png]
